# Arsenic Methylation and its Relationship to Abundance and Diversity of *arsM* Genes in Composting Manure

**DOI:** 10.1038/srep42198

**Published:** 2017-03-07

**Authors:** Weiwei Zhai, Mabel T. Wong, Fei Luo, Muhammad Z. Hashmi, Xingmei Liu, Elizabeth A. Edwards, Xianjin Tang, Jianming Xu

**Affiliations:** 1 Institute of Soil and Water Resources and Environmental Science, College of Environmental and Resource Sciences, Zhejiang Provincial Key Laboratory of Agricultural Resources and Environment, Zhejiang University, Hangzhou 310058, China; 2 Department of Chemical Engineering and Applied Chemistry, University of Toronto, Toronto, M5S 3E5, Canada; 3 Department of Meteorology, COMSATS Institute of Information Technology, Islamabad Campus, Park Road, Chak Shahzad, Islamabad, Pakistan

## Abstract

Although methylation is regarded as one of the main detoxification pathways for arsenic (As), current knowledge about this process during manure composting remains limited. In this study, two pilot-scale compost piles were established to treat manure contaminated with As. An overall accumulation of methylated As occurred during 60 day-composting time. The concentration of monomethylarsonic acid (MMA) increased from 6 to 190 μg kg^−1^ within 15 days and decreased to 35 μg kg^−1^ at the end of the maturing phase; while the concentration of dimethylarsinic acid (DMA) continuously increased from 33 to 595 μg kg^−1^ over the composting time. The *arsM* gene copies increased gradually from 0.08 × 10^9^ to 6.82 × 10^9^ copies g^−1^ dry mass over time and correlated positively to the concentrations of methylated As. 16S rRNA gene sequencing and *arsM* clone library analysis confirmed the high abundance and diversity of *arsM* genes. Many of these genes were related to those from known As-methylating microbes, including *Streptomyces sp., Amycolatopsis mediterranei and Sphaerobacter thermophiles*. These results demonstrated that As methylation during manure composting is significant and, for the first time, established a linkage between As biomethylation and the abundance and diversity of the *arsM* functional genes in composting manure.

The rapid expansion of the poultry and livestock industries in the past decades has generated vast quantities of farming waste, with attendant environmental impacts, notably in farming-intensive countries such as China, India and Brazil^1^,^2^,^3^. For instance, ~2.2 billion tons of poultry and livestock manure were generated in China in 2011 alone^4^. The manure usually contains a large amount of nutrients, inorganic and organic contaminants, antibiotic resistance genes, and pathogens, most of which are potential sources of pollution and pose risks for the environment^5^. Composting is an economical and environmentally friendly approach for reducing and attenuating manure waste^6^, and has been widely used in China and other countries around the world. Currently, the behavior and biotransformation of inorganic and organic pollutants during composting of livestock waste are major research focus.

Arsenic (As) is a potent environmental toxin and human carcinogen^7^ that is linked to increased risk of bladder, lung, and skin cancers^8^ and ranks the top in the list of hazardous substances by US Environmental Protection Agency (EPA). Despite its toxicity, As-based feed additives are commonly used in the poultry and livestock industry to prevent disease, enhance feed efficiency and promote rapid growth^9^. Not readily absorbed in animal tissues, almost all the fed As is excreted without attenuation in manure at concentrations up to 300 mg kg^−1^ 9,10. In nature, As exists in inorganic and organic forms such as arsenate [As(V)], arsenite [As(III)], monomethylarsonic acid [MMA], dimethylarsinic acid [DMA], trimethylarsinic acid [TMA] and trimethylarsine oxide [TMAO] with varying biogeochemical behaviors and toxicity^11^. Methylated As species have been found in soils, but as minor species compared to inorganic As^12^,^13^. Methylated As species, mostly in the form of DMA, as well as MMA and tetramethylarsonium, have been reported in rice grains which in trace amounts originated from soils^14^,^15^,^16^. Methylated As species, both volatile (e.g. TMA and TMAO) and non-volatile (e.g. MMA and DMA), are less toxic than their inorganic forms^17^. Therefore, methylation of As is normally regarded as one of the main detoxification pathways for As in the environment^18^.

Many studies that explore As methylation during composting have been published recently^18^, driven by the incentive to detoxify As in wastes. Diaz-Bone *et al*. reported that metal(loid)s could undergo intensive biomethylation during the composting of As hyperaccumulating fern *Pteris vittata*^19^. Maňáková *et al*. observed a slight decrease in DMA and MMA contents during the composting of waste sludge^20^. However, the microbial dynamics of As methylation and resulting speciation during composting of pig manure remains to be explored.

The stepwise microbial methylation of inorganic arsenite is catalyzed into its methylated counterparts (e.g. [As(III)]→[MMA]→[DMA]→[TMA]) by S-adenosylmethionine methyltransferase encoded by *arsM* genes^18^. Since its first isolation from soil bacterium *Rhodopseudomonas palustris*^18^, *arsM* has been identified in many other microbes, including *Pseudomonas spp*.^21^, methanogens^22^, *Halobacterium sp. NRC-1*^23^ and a number of eukaryotic algae^24^. Recently, *arsM* was identified even more in phylogenetically-diverse microbial communities including *Actinobacteria, Gemmatimonadales, α-Proteobacterales, β-Proteobacterales, δ-Proteobacterales, Firmicutes, Archaea*, and other organisms residing in rice rhizosphere soil and roots^25^. The function of the proteins encoded by the *arsM* genes was first demonstrated in an As-hypersensitive strain of *Escherichia coli*, where recombinant expression of an *arsM* gene conferred As resistance^26^. Similarly, transgenic rice expressing an *arsM* gene from *R. palustris* was shown to methylate inorganic As into a variety of organic As species^27^. Although the mechanism of microbial As methylation is known and *arsM* genes have been detected in various environments, there remains a limited understanding of how the abundance and diversity of *arsM* genes correlate with the methylation process during manure composting.

In this study, two pilot-scale pig manure composting piles were constructed for a systematic investigation of As methylation. Physical and chemical parameters of the piles were monitored during composting process. Microbial community composition and abundance, as well as the abundance and diversity of *arsM* genes were monitored using real-time PCR (qPCR) and amplicon sequencing. The results have yielded the first clear connection between As methylation and microbial *arsM* gene abundance in manure composting, providing valuable insights for developing strategic management of manure waste.

## Results

### Physical-chemical properties and total As changes during composting

Two pilot-scale pig manure compost piles (MC1 and MC2) were established and monitored over 60 days. Based on the measured temperature profile, both compost piles progressed through mesophilic (day 0~4), thermophilic (day 5~42), and maturing phases (day 43~60) as defined in other composting processes^28^. A rapid increase in temperature from ~31 °C to ~60 °C within the first 4 days was observed (Supplementary Figure S1), and this high temperature was maintained throughout the thermophilic phase for approximately 42 days before dropping to ~40 °C in the subsequent maturing phase. Over the composting period, samples were collected to track their activity. The organic matter (OM) content decreased gradually from ~60% to ~41% (g VS/g dry mass) after 60 days, and the moisture content decreased from ~64% to ~43% in the composting piles (Supplementary Table S1). In the first 15 days, the pH decreased from 6.9 to 3.5 and then gradually increased back up to around 6.0. Bulk oxidation-reduction potential (*Eh)* decreased from 174 mV to 60–65 mV after 60 days and NH_4_^+^-N concentrations also decreased substantially as the composting proceeded (Supplementary Table S1). The total As content on a per kg dry solids basis increased from 1,270 ± 62 μg kg^−1^ to 1,800 ± 68 μg kg^−1^ in MC1 and from 1,240 ± 67 μg kg^−1^ to 1,720 ± 180 μg kg^−1^ in MC2 over time, amounting to a ~1.4 fold increase in concentration in both compost piles by day 60 (Supplementary Figure S2).

### The change of As species during composting

**Figure 1:**
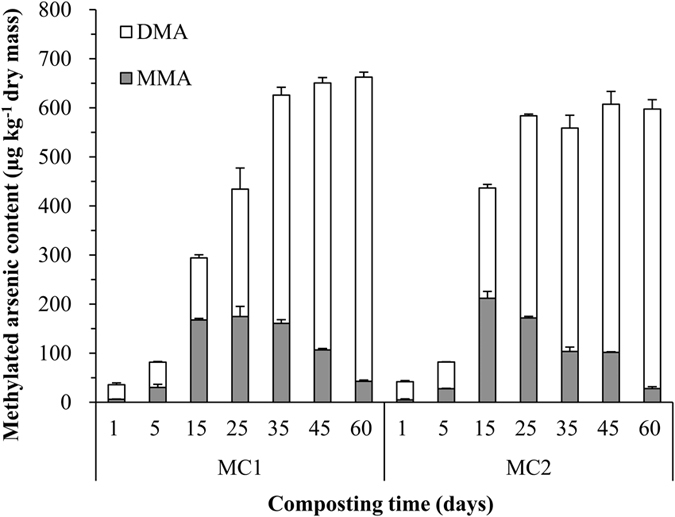
Changes in concentrations of methylated As (MMA and DMA) in the two compost piles. Error bars represent the standard error of 3 replicate analysis of a composite sample. A composite sample was made up of 10 subsamples from different locations in compost piles. The composting time was divided into mesophilic (day 0–4), thermophilic (day 5–42), and maturing phases (day 43–60).

The concentrations of two major methylated As species, MMA and DMA, were shown in Fig. 1. MMA content in both compost piles peaked during the thermophilic phase (day 5–42) and dropped rapidly during the maturing phase (day 43–60). The MMA concentrations in MC1 peaked at 175 ± 21 μg kg^−1^ at the middle of the thermophilic phase (day 25) and dropped subsequently rapidly to 43 ± 3 μg kg^−1^ in the maturing phase (day 60). The MMA concentrations in MC2 exhibited a similar trend, peaking at 222 ± 14 μg kg^−1^ on day 15, and subsequently dropping from day 15 onwards to a final concentration of 28 ± 4 μg kg^−1^ on day 60. In contrast, DMA concentrations increased steadily in both compost piles over the composting period (Fig. 1), from 30 ± 4 to 620 ± 10 μg kg^−1^ in MC1 and from 37 ± 3 to 570 ± 20 μg kg^−1^ in MC2. The total concentrations of methylated As species increased more rapidly during the mesophilic and thermophilic phases, while only small increases during the maturing phase. Moreover, there were no significant differences in the concentrations of methylated As species at most sampling times between the two composting piles (MC1 and MC2) as determined using a two-way ANOVA test (Supplementary Table S2).

### Copy numbers of bacterial 16S rRNA and *arsM* genes

**Figure 2:**
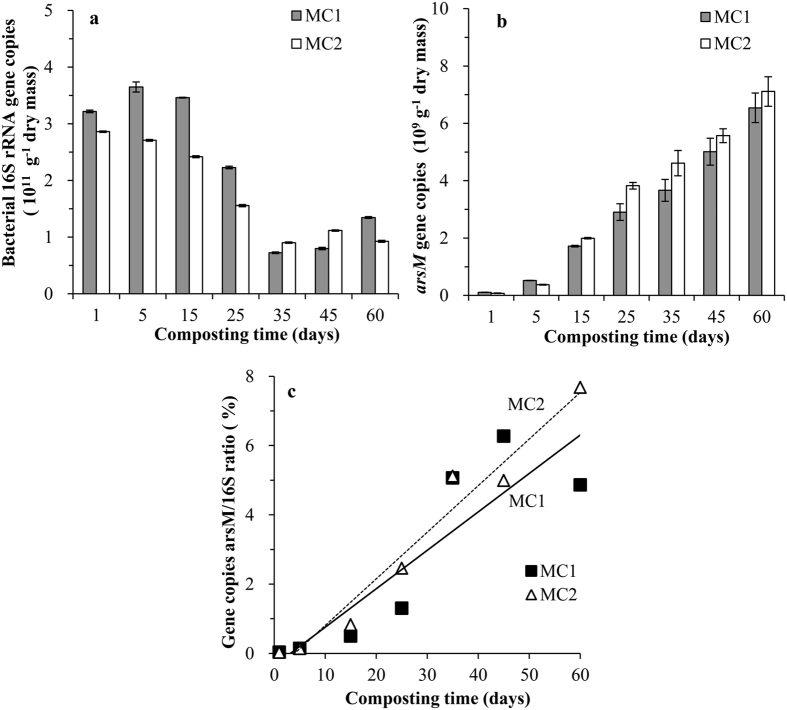
Plot of 16S rRNA gene copies (**a**), *arsM* gene copies (**b**) and ratio of *arsM*/16S rRNA (**c**) in the two compost piles. Lines of best fit are shown in panel c illustrating increasing proportion of organisms containing *arsM* gene with time.

**Figure 3:**
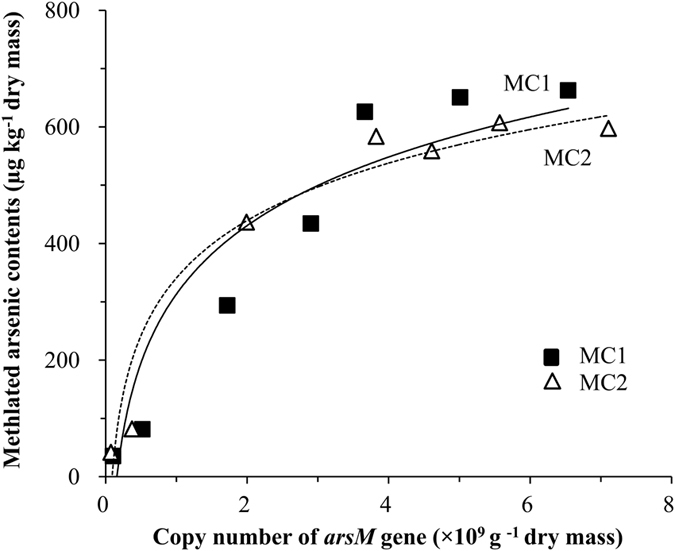
Plot of methylated As concentration versus *arsM* copies in two compost piles. The symbols represent experimental data and the curves provide a logarithmic fit.

The copy numbers of the bacterial 16S rRNA gene (copies g^−1^ dry mass) in the compost piles during the experimental period is shown in Fig. 2a. The abundance of 16S rRNA genes decreased from ~3 × 10^11^ to 0.8 × 10^11^ copies g^−1^ dry mass during the thermophilic phase. The abundance of *arsM* genes in MC1 and MC2 as a function of composting time increased gradually from ~0.1 × 10^9^ to ~6.8 × 10^9^ copies g^−1^ dry mass in both piles (Fig. 2b). There were also no significant differences in *arsM* gene copies between MC1 and MC2 except for those samples on day 25 and 35 (Supplementary Table S2). Significantly, a steady and significant increase in the ratio of *arsM* genes to 16S rRNA gene numbers was observed. The highest value (~6% on day 45, MC1; ~8% on day 60, MC2) occurred during the maturing phase (Fig. 2c). Further, the sum of MMA and DMA concentrations at different time points was found to correlate strongly with the *arsM* gene copy numbers (Fig. 3).

### Dynamics in microbial community structure

The V6-V8 region 16S rRNA gene was amplified and sequenced, returning an average of ~23,000 reads per sample. All samples showed relatively high coverage (0.74–0.89) and high diversity as indicated by Shannon, ACE, and Chao1 indices (Supplementary Table S3). A diverse set of OTUs from 18 orders from 14 phyla were detected in MC1 and MC2 with major shifts over the composting period (Supplementary Figure S3). The relative abundance of *Firmicutes* decreased markedly while significant increases in the proportions of *Actinobacteria, Proteobacteria and Bacteroidetes* were observed (Supplementary Figure S3). At the beginning of the composting period, *Firmicutes* accounted for ~93% of the total population; by the end, they accounted for only 46.8% (MC1) and 41.2% (MC2). As shown in Supplementary Figure S3, the relative abundance of *Actinobacteria, Proteobacteria* and *Bacteroidetes* at the end were 18.7%, 9.4% and 16.0% in MC1, and 30.0%, 9.6% and 4.5% in MC2, respectively. The relative abundance of *Chloroflexi* increased from 0.04% to 1.4% in MC1 and 0.01% to 3.2% in MC2 over the composting process, respectively. Considering the OTU sequences clustered at the order level, the samples from MC1 and MC2 shared similar profiles over time (Supplementary Figure S3). At the beginning of the composting, *Clostridiales, Lactobacillales* and *Erysipelotrichales* were dominant, accounting for ~90% of the total population. *Halanaerobiales* and *Bacillales* significantly increased within the first 15 days, and then decreased markedly, while *Flavobacteriales* and *Burkholderiales* increased in the maturing phase. *Pseudonocardiales* and *Sphaerobacterales* were not dominant in the initial microbial community structure (0.25% and 0.65%), however, they both increased during the thermophilic and maturing phases and increased to ~1–2% of the population by day 60, respectively (Supplementary Figure S3).

**Figure 4:**
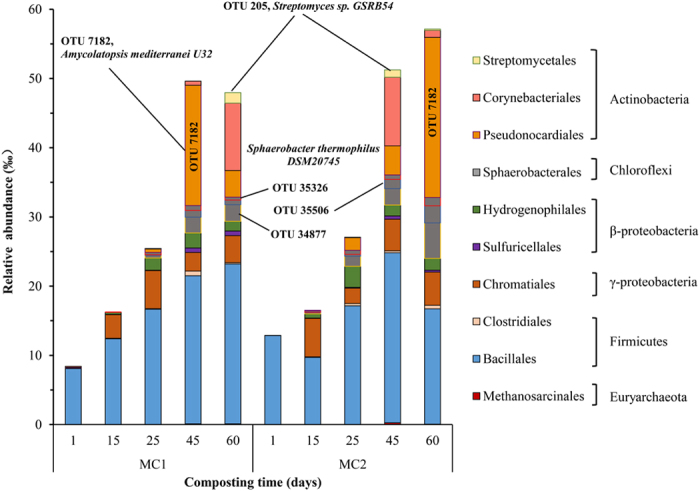
Changes and taxa of selected 16S OTUs related to As methylation. A custom database of microorganisms contained *arsM* genes was constructed by compiling all 16S rRNA gene sequences from NCBI. All 16S rRNA gene sequences of samples were checked against the database, and sequences that have a similarity ≥95% and Read depth ≥10 were retained. Changes of these OTUs with composting time are shown. Note abundance is in per mil, not percent. Maximum abundance is about 6%, which is similar to the max ratio of *arsM* to 16S rRNA genes recovered (see Fig. 2c). Refer to Supplementary Table S4 for specific OTUs per group.

In order to gain more specific knowledge about organisms responsible for As methylation, we aligned the 16S rRNA sequences from the compost piles against 16S rRNA sequences from microbes containing an *arsM* gene (Supplementary Table S5). Eighty-three OTUs from the compost piles match to previously report hosts of arsM genes (similarity ≥ 95%) and their relative abundance clearly increased with composting time (Fig. 4). At the beginning of composting, these matching OTUs were primarily *Bacillales* within the *Firmicutes* and made up only 0.8 to 1.3% of the total population (note: Y-axis in Fig. 4 is in per mil, not percent). *Bacillales, Hydrogenophilales* and *Chromatiales* all increased in relative abundance over time. *Pseudonocardiales* and *Corynebacteriales* were also relatively highly represented in the samples. *Sphaerobacterales (Chloroflexi)* increased to 0.4% (MC1) and 0.9% (MC2) on day 60. *Streptomycetales* could only be detected on day 60 (0.15% in MC1 and 0.01% in MC2).

**Figure 5:**
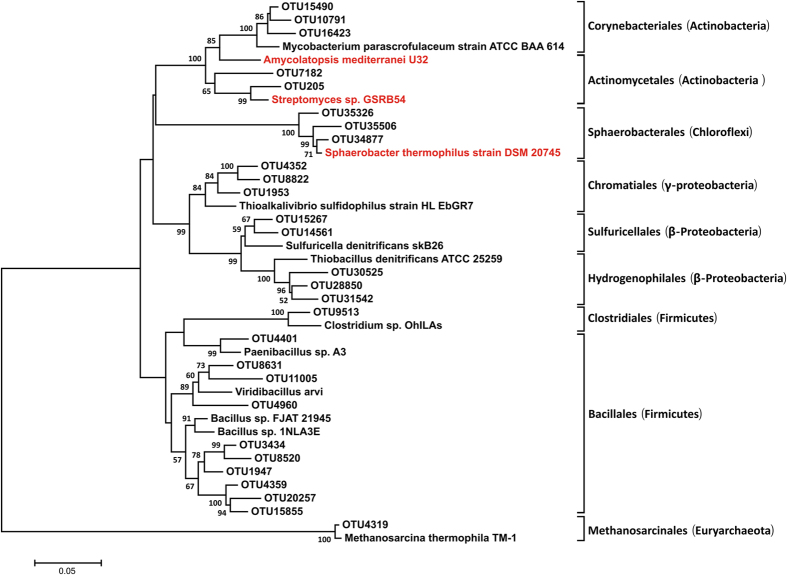
Neighbor-joining analysis of representative 16S OTUs obtained from the composting samples using MEGA 6.05. A custom database of microorganisms contained *arsM* genes was constructed by downloading corresponding 16S rRNA gene sequences from NCBI. All 16S rRNA gene sequences from composting samples were checked against this database, and sequences that have a similarity ≥95% and read depth ≥10 were retained. Bootstrap values >50% are shown on nodes. The scale bar indicates sequence dissimilarity between nodes. The taxonomic assignment of OUTs of the compost piles is indicated in parentheses.

A neighbor-joining tree of 27 representative 16S OTUs from the total 83 OTUs matching to 16S rRNA sequences of known *arsM*-containing organisms was shown in Fig. 5. The most abundant OTU (OTU7182) was closely related to *Amycolatopsis mediterranei* U32 (95% similarity). OTU4960 and OTU1947 were respectively similar to *Viridibacillus arvi* (95%) and *Bacillus sp. FJAT-21945* (97%). OTU20257, OTU4359, OTU15855 were all similar to *Bacillus sp. FJAT-21945* (95%). OTU34877 was nearly identical to *Sphaerobacter thermophiles DSM20745* (99%). OTU1953 and OTU4352 were affiliated with *Thioalkalivibrio sulfidophilus HL-EbGr7* (96%; 95%) and OTU205 clustered near *Streptomyces sp. GSRB54* (97%). OTU28850 was similar to *Thiobacillus denitrificans ATCC 25259* (95%). The relative abundance of OTU 205 (*Streptomyces sp*.), OTU7182 (*Amycolatopsis mediterranei*), OTU34877, OTU35506, OTU35326 (*Sphaerobacter thermophiles*) along with composting time were also shown in Fig. 4. Complete abundance data and similarities were provided in Supplementary Table S4.

### The abundance and biodiversity of *arsM* genes

**Figure 6:**
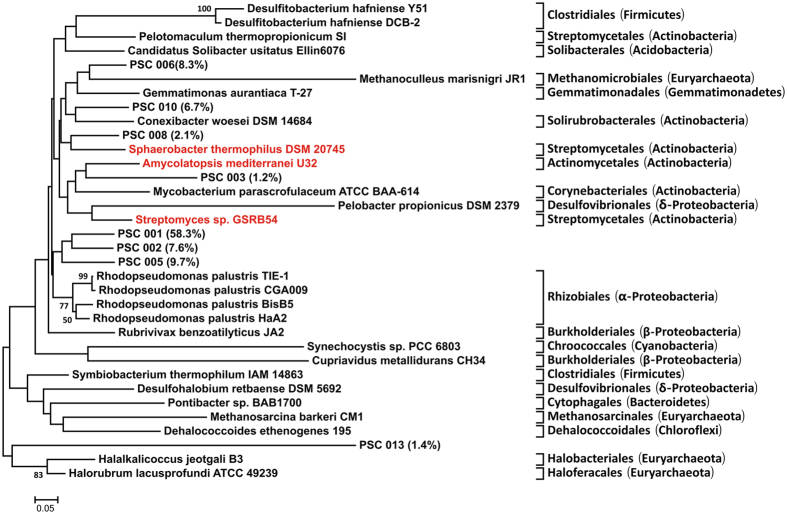
Neighbor-joining analysis of *arsM* sequences retrieved from composting samples using MEGA 6.05. Numbers in brackets after partial sequence clones (PSCs) number indicate relative abundance in the clone library. Only sequence representatives with an 89% nucleotide similarity to PSCs cutoff are shown in tree. Bootstrap values >50% are shown on nodes. The scale bar indicates sequence dissimilarity between nodes. The taxonomy note shown at the right is based on known sequences and corresponding species.

The *arsM* genes encoding S-adenosylmethionine methyltransferase were amplified from DNA samples collected on day 15 and day 60 using previously published primers^25^ targeting most known *arsM* genes. Resulting clone libraries were sequenced to assess the diversity of these genes. Sequences were clustered and results were visualized in a heat map (Supplementary Figure S4). This sequencing data confirmed that a high abundance and diversity of *arsM* genes could be found in compost piles. The most abundance partial sequence clones (PSCs) were PSC001, accounting for 58.3% of total clones. Besides, PSC002 (7.6%), PSC003 (1.2%), PSC005 (9.7%), PSC006 (8.3%), PSC008 (2.1%), PSC010 (6.7%), PSC013 (1.4%) were also abundant. PSC001, PSC008 and PSC013 were most abundant in the samples collected on day 15, while PSC005, PSC002, PSC010 and PSC003 were most abundant in those samples collected on day 60. PSC006 was abundant across all samples. All PSCs were further compared with the database of referenced *arsM* gene sequences from NCBI (Supplementary Table S5). A Neighbor-joining tree of the 8 most abundant *arsM* PSCs (>1% relative abundance) was constructed with selected references (Fig. 6). PSC001, PSC002 and PSC005 did not match closely to any known sequences and thus could not be associated with a phylogenetic group. However, PSC006 was affiliated with *Methanoculleus marisnigri JR1*, and PSC010 were similar to *Conexibacterwoesei DSM 14684*. In addition, PSC006 and PSC010 clustered near the *arsM* gene from *Gemmatimonas aurantiaca T27*. PSC003 was associated with *arsM* gene from *Amycolatopsis mediterranei U32, Mycobacterium parascrofulaceum ATCC BAA-614, Pelobacter propionicus DSM 2379 and Streptomyces sp. GSRB54.* PSC008 were nearly identical to the *arsM* sequences found in *Sphaerobacter thermophilus DSM20745*. PSC013 was categorized near the *arsM* gene from *Halalkalicoccus jeotgali B3 and Halorubrum lacusprofundi ATCC 49239*. In addition, many of rare *arsM*-like PSCs (relative abundance <1%; not shown) were associated with *Rhodopseudomonas palustris TIE1, Rhodopseudomonas palustris CGA009, Streptomyces sp. GSRB54, Rubrivivax benzoatilyticus JA2, Symbiobacterium thermophilum IAM 14863.*

## Discussion

The rapid and sustained increase of temperature in the thermophilic stage in the two compost piles is an indicator of rapid establishment of microbial activity (Supplementary Figure S1), as biodegradation of organic matter resulted in a substantial heat production^20^,^29^. During the manure composting, As became more concentrated in the compost (Supplementary Figure S2), which was consistent with bulk mass loss via respiration and mineralization of organic matter during composting^30^. Song *et al*. reported that As concentrations increased by 57% in cow manure compost and 36% in pig manure compost^31^. However, the total As concentration is an overall pollution indicator but provides little information about which species are present, or about their potential of mobility and bioavailability in the environment. The total concentrations of methylated As species increased in the composting piles especially during the mesophilic and thermophilic phases. The concentrations of methylated As species represented 37% (MC1) and 35% (MC2) of total As concentration by day 60, clearly indicating As methylation during manure composting. Similarly, the conversion rates up to 50% for As have been reported in alfalfa hay composting^19^. Methylated As species analysis also showed that MMA concentrations peaked in the thermophilic phase and dropped subsequently rapidly in the maturing phase (Fig. 1). However, a continuous increase was found in DMA concentrations, indicating the transformation of MMA to DMA (Fig. 1). Diaz-Bone *et al*. reported that the concentrations of methylated species, first MMA, then DMA, peaked during the thermophilic phase of dry alfalfa hay composting, while at the end of the thermophilic phase both MMA and DMA decreased^19^. Higher temperature could considerably accelerate the rate of As methylation by enhancing microbial or enzymatic activities. Algal enzymes were shown to convert As(III) to DMA within 30 min at 70 °C, but not at 37 °C^18^. Purified recombinant CmArsMs enzymes were shown to transform As(III) into MMA, DMA and TMAO with an optimum temperature of 60–70 °C^18^. Bas Vriens *et al*. reported an increased rate of As methylation with increasing temperatures in both surface water and air suggesting that the methylation of As is temperature dependent^32^. It is also reported that at high pH, the presence of organic matter and moderate moisture and temperature would favor microbial-mediated As biotransformation^33^. Methylated As concentrations were found to correlate significantly negatively with soil pH, but positively with dissolved organic carbon^34^. Furthermore, the addition of organic matter stimulated the methylation of As species^35^,^36^. A recent study has shown that As methylation increased with the decrease of *Eh* values in a soil environment^37^. Thus, the lower *Eh* values in the maturing phase (shown in Supplementary Table S1) may have also enhanced As methylation during manure composting in this study. The stepwise As methylation was indicated by successive maxima of methylated As, while continuously increase of DMA was observed, TMA was not detected in the current study. The conversion of DMA to TMA is the rate limiting step in As methylation^38^. Therefore, DMA commonly accumulates in the environment. In the present study, the total concentrations of methylated As species increased little in maturing phases , reflecting the balance between As methylation and the losses of methylated As. MMA transformed to DMA and a fraction of DMA may be reduced to dimethylarsine, or further methylated to form trimethylarsine, both of which are volatile. It was reported that up to 35% of As was volatilized from anaerobic digestion of cow dung using cultures of methanogenic bacteria^39^. Moreover, As can be volatilized into methylarsines, mainly trimethylarsine and some mono- and dimethylarsine in model biogas digesters when the major substrates were rice straw and animal manure^40^. Therefore, we postulate that a portion of As in the manure volatilized into the atmosphere during composting in our study and the risk of the volatile As in the compost should be paid more attention. However, the species and levels of volatile As in this experiment remains unknown, which will be determined in our further studies.

Methylation of As is mediated by microbes and *arsM* gene plays an important role. The *arsM* gene copies has been investigated mostly in soil in previous studies, For example, Jia *et al*. found that the copy numbers of *arsM* genes in paddy soil were in the order of 10^7^–10^8^ copies g^−1^ dry soil assessed by qPCR assay^25^. In the present study, the copy numbers of the *arsM* gene (Fig. 2b) indicated that the As methylation potential could be highly detected in composting samples. The increase of *arsM* gene copies with composting time was consistent with other studies. Zhao *et al*. reported that the *arsM* gene copy numbers correlated positively with soil pH^34^. Carbonell-Barrachina *et al*. also found that As methylation could be measurable at pH 6.5 and 8.0 in a sewage sludge suspension, but was drastically restricted at pH 5.0^41^. In our study, the pH values of our compost piles gradually increased to 6.0 on average, and *Eh* gradually decreased to 62.0 mV on average from day 15 in both compost piles (Supplementary Table S1), which stimulated As methylation. Furthermore, the high concentration of organic substrates in manure may increase carbon and energy sources for the As methylating microbes, which in turn has a positive effect on As biomethylation^36^. The copy numbers of 16S rRNA gene decreased during the thermophilic phase and rebounded slightly in the maturing phase (Fig. 2a), which was similar to the results reported by Wang *et al*.^42^. This suggested that the temperature may highly affect the microbial community structure dynamics during manure composting^43^,^44^. In our case, the proportion of *arsM*-containing microbes increased steadily over the course of compositing, as shown by the progressive increase in the ratio of *arsM* genes copy numbers to bacterial 16S rRNA gene copy numbers over time (Fig. 2c). Thus *arsM*-harboring microbes constituted a substantial (~5–8%) fraction of the bacterial community in the later phase of composting. At the same time, the concentrations of methylated As correlate positively with the copy numbers of *arsM* genes (Fig. 3). Ma *et al*. reported a positive relationship between *arsM* gene copy numbers and the concentrations of organic As in rice grain^45^. Jia *et al*. also found a positive linear relationship between *arsM* gene copy numbers and concentrations of methylated As species in a soil solution of rhizosphere and bulk soils^25^. Considering the *arsM* genes play the key roles in microbial As methylation^18^,^46^, the increasing ratio of *arsM* gene copies to bacterial 16S rRNA gene copies along with the composting process and the positive correlation between the concentrations of methylated As with the copy numbers of *arsM* genes confirmed the As methylation ability in the manure compost piles. The ratio of *arsM* to bacterial 16S rRNA gene copies appears to be a very useful biomarker and predictor of As methylation in many different systems. Moreover, both compost piles MC1 and MC2 behaved similarly (Supplementary Table S2), further confirming our results.

Much attention has been paid to the bacterial communities during the composting process. *Firmicutes,* represented primarily by *Bacilli*, were reported as a major group in the mesophilic- and thermophilic-phases of coffee composting and the microbial diversity was significantly limited by temperature^47^,^48^. In our study, the overall abundance of *Firmicutes* decreased (Supplementary Figure S3), but *Firmicutes* was dominant and increased with composting time in the analysis of 16S OTUs related to As methylation (Fig. 4). This suggested that some *Firmicutes* might be key microbial members contributing to As methylation in compost piles. Some *Firmicutes* have been reported to harbor putative As resistance genes^49^. *Actinobacteria* (containing abundant orders *Pseudonocardiales, Streptomycetales*, and *Corynebacteriales*) were also found to increase subsequently as the compost pile entered the maturing phase (Fig. 4), as reported in other compost sites^50^. *Firmicutes, Actinobateria*, and *Proteobacteria* were prevalent in high As aquifers and acid mine drainage^51^,^52^ suggesting these microbial members are As resistant.

Further, phylogenetic analysis of representative 16S OTUs revealed the abundance of species likely responsible for As methylation in the compost samples (Fig. 5). For example, As biomethylation has also been observed in anaerobic archaea, such as methanogens. We identified an OTU (OTU4319) similar to *Methanosarcina thermophila TM-1* in samples from maturing phases. *Streptomyces sp. strain GSRB54*, a bacterium responsible for As methylation, was isolated from the roots of rice plants grown in As-contaminated paddy soil under anaerobic conditions. An OTU (OTU205) similar to *Streptomyces sp. strain GSRB54*, was also found to be more abundant in the samples from the maturing phases, when a higher methylated As content was present (Supplementary Table S4). We also identified OTUs similar to *Sphaerobacter thermophilus DSM20745* whose *arsM* gene has been detected in soil^34^. *Amycolatopsis mediterranei* has been used to examine multiple alignments of deduced amino acid sequences of microbial *arsM*^53^. These results indicated that 16S OTUs similar to these strains might have an association with As methylation.

Phylogenetic analysis of *arsM* clones showed high diversity of *arsM* genes in the composting samples (Fig. 6). The function of the *arsM* genes was clearly established when it was cloned from *Rhodopseudomonas palustris* and expressed in an arsenic-hypersensitive strain of *Escherichia coli*^18^. *Rhodopseudomonas palustris* has also been a good model organism for studying As detoxification^54^. *Clostridiales, Streptomycetales, Desulfovibrionales* and *Burkholderiales*, have also been found to be widely distributed in an As polluted river^55^; these same groups were also revealed from *arsM* gene sequences in our compost piles. However, the most abundant *arsM* partial sequence clones (PSCs), PSC001, PSC002 and PSC005 belonged to unknown taxa indicating *arsM* diversity begging further investigation. We also identified PSCs with high similarity to *arsM* genes from *Streptomyces sp., Amycolatopsis mediterranei* and *Sphaerobacter thermophiles.* Meanwhile, *Streptomyces sp. and Amycolatopsis mediterranei* classified in the *Actinobacteria* phylum were also found in the phylogenetic analysis of 16S OTUs (Fig. 5). The correspondence between 16S rRNA and *arsM* phylogeny, and increases in the abundance during composting confirm that *Streptomyces sp., Amycolatopsis mediterranei and Sphaerobacter thermophiles* might be highly involved and more active than other microbes in mediating As methylation during manure composting. Besides, the increased abundance of *Actinobacteria* in the analysis of 16S OTUs related to As methylation further suggested that *Actinobacteria* played an important role in the methylation process. However, further studies should be carried out to verify whether these species were the key contributors to As methylation during manure composting.

Furthermore, there remains a discrepancy between the 16S rRNA gene sequencing and *arsM* clone library analysis results. For the two most abundant 16S OTUs shown in Supplementary Table S4, OTU4960 (similar to *arsM*-containing *Viridibacillus arvi*) and OTU1947 (similar to *arsM*-containing *Bacillus sp. FJAT-21945*), we could not identify the corresponding *arsM* gene in our clone analysis (Fig. 6). Conversely, in the *arsM* clone analysis, beside *Rhodopseudomonas palustris, arsM* genes from *Pelotomaculum thermopropionicum, Rubrivivaxbenzoatilyticus*, and *Gemmatimonasaurantiaca* could not be found in the 16S rRNA gene phylogeny (Fig. 5) and the most abundance PSCs could not be assigned to a known phylogeny. This suggested a good genetic diversity of *arsM* gene, however, we still don’t know exactly what taxa PSC001, PSC002 and PSC005 are from. On the other hand, our results also suggested the inconsistency of 16S rRNA gene sequencing and *arsM* clone library analysis. There are possible reasons for this inconsistency. Firstly, Depth of sequencing and primer biases may explain some of these discrepancies. It is more likely that the actual distribution and diversity of *arsM*-containing microbes is much broader than currently known. Secondly, prokaryotes (and some eukaryotes) are asexual and could not form species in a genetic way. Thirdly, horizontal gene transfer contributed to difficulties in assigning phylogenty, also reported by Jia *et al*. in soil and rice rhizosphere^25^. To date, environmental diversity surveys of As methyltransferase *arsM* genes remain limited to paddy soils and rice rhizosphere identifying many putative *arsM*-containing genera, including *Actinobacteria, Gemmatimonadales, α-Proteobacterales, δ-Proteobacterales, β-Proteobacterales, Sphaerobacterales, Firmicates*, CFB group bacteria, *Halobacteriales*, Archaea and other 5 unknown groups^25^. Zhang *et al*. reported that *arsM* sequences in paddy soils belonged to *Gemmatimonadales* (16%), *Firmicutes* (9%), *Actinobacteria* (11%), *Proteobacteria* (34%), and *Archaea* (6%)^56^. The present study showed putative *arsM* affiliated with a wide range of phylogenetic taxa that were present in all composting samples. The wide distribution of As(III) methyltransferases during composting indicated the potential for As methylation resulting in the accumulation of methylated As species. The abundance and diversity of *arsM* suggested *arsM*-carrying bacteria detected by limited molecular approaches may be generalists and As methylation process in pig manure composting may have been overlooked. Therefore, comprehensive analysis or a combination of different analysis should be used for studying the functional microbes in the composting.

In conclusion, two pilot-scale pig manure compost piles were established and an accumulation of methylated As was revealed over the composting process. By qPCR, 16S rRNA sequencing and clone libraries, a diverse group of *arsM* genes in composting pig manure have been confirmed presented in high abundance and diversity, and they increase along with the composting process. *Streptomyces sp., Amycolatopsis mediterranei and Sphaerobacter thermophiles* were found in both 16S rRNA and *arsM* gene sequence libraries, suggesting that these bacteria might be highly involved in the As methylation during manure composting. The inconsistency of 16S rRNA gene sequences and *arsM* clone library sequences betray a wider diversity than currently appreciated. To our knowledge, this is the first exploration of As methylation and the diversity and distribution of *arsM* gene during manure composting. Moreover, quantitative analysis of the ratio between *arsM* to bacterial 16S rRNA gene copies appears to be a very good indicator of As methylation in many different systems. Further work should be conducted to define the connection between phylogeny and *arsM* gene clone sequence and activity.

## Materials and Methods

### Composting experiments and sampling

Two independent pilot-scale (2.5 m × 1.8 m × 0.75 m in length, width and height) pig manure compost piles were set up in Hangzhou, China, containing on average 1750 ± 60 μg kg^−1^ As (dw, n = 3). The first manure compost pile (MC1) was composed of 1,200 kg pig manure and 600 kg sawdust for optimal C/N ratio and water content. The second manure compost pile (MC2) contained 1,200 kg pig manure and 600 kg sawdust mixed with burned rice straw. The moisture contents of the composting sites were maintained at approximately 65% by sprinkling water once every two days. Furthermore, compost piles were turned over and mixed once every two days in the first month, and once every four days in the second month for aeration. The whole composting process lasted for 60 days, and 2 kg samples were collected on day 1, 5, 15, 25, 35, 45 and 60. Each time, samples representative of the compost piles were generated by mixing equal portions of compost samples collected from the surface (20 cm to top, 20 cm from side), center, and bottom (20 cm from bottom, 50 cm from side) Sample properties, including temperature, pH, *Eh*, total C/N ration, OM, moisture content, NH_4_^+^-N concentration and total As content were determined. Concentrations of methylated As species, MMA and DMA, were measured by High-Performance Liquid Chromatography Coupled with Inductively Coupled Plasma Mass Spectrometry (HPLC-ICP-MS, NEXION300XX, PerkinElmer, Inc., USA). Details are shown in the Supporting Information. The remaining composts from the chemical analysis were stored immediately at −20 °C.

### Determination of bacterial 16S rRNA and *arsM* genes copy numbers by qPCR

Compost samples were thawed and total DNA was extracted using the FastDNA SPIN Kit for Soil (MP Biomedicals, USA) according to the manufacturer’s protocol. The extracted DNA was then quantified using a Nanodrop ND-2000 UV-Vis spectrophotometer (NanoDrop Co., USA) and stored at −20 °C until further analysis. Copy numbers of bacterial 16S rRNA gene in the compost samples were estimated by qPCR using primers 1369F (5′-CGGTGAATACGTTCYCGG) and 1492R (5′-GGWTACCTTGTTACGACTTT). The *arsM* genes were recovered using universal *arsM* gene primers as described previously^25^. qPCR assays of the *arsM* genes used the primers *arsM*F (5′-TCYCTCGGCTGCGGCAAYCCVAC) and *arsM*R (5′-CGWCCGCCWGGCTTWAGYACCCG). Details of the amplification conditions of bacterial 16S rRNA and *arsM* genes are in the Supporting Information.

### 16S rRNA gene sequencing

The V6-V8 hypervariable region^57^ 16S rRNA gene was amplified using universal primers 926F (AAA CTY AAA KGA ATT GAC GG) and 1392R (ACG GGC GGT GTG TRC) with the incorporation of multiplex barcodes^58^. The 16S rRNA gene PCR amplifications were then sent for sequencing using Illumina Miseq sequencing platform (Miseq, Illumina Inc., USA) at Zhejing Institute of Microbiology, China. The details of the 16S rRNA gene PCR, sequencing and bioinformatic analysis are presented in the Supporting Information. Further, a database of microorganisms containing an *arsM* gene was constructed by downloading all 16S rRNA and corresponding *arsM* gene sequences from NCBI (Supplementary Table S5). All 16S rRNA gene OTUs of samples were checked against the database and 83 sequences that have a nucleotide similarity ≥95% and read depth ≥10 were retained.

### Construction of the *arsM* Clone Library

Four samples (15-MC1, 15-MC2, 60-MC1 and 60-MC2) were selected for the construction of *arsM* gene clone libraries. The *arsM* genes were recovered using universal *arsM* gene primers *arsM*F and *arsM*R as described previously^25^. Details of the amplification conditions and the *arsM* sequence analysis are in the Supporting Information. A total of 23 different *arsM* partial sequence clones (PSCs), ≥89% similarity level, were recovered from 422 sequenced clones using the MOTHUR program^59^,^60^. All *arsM* PSCs were checked against the database shown in Supplementary Table S5.

### Phylogenetic analysis

27 representative 16S rRNA OTUs from 83 retained 16S OTUs and 13 representative 16S rRNA gene sequences of each order from the database were selected and a neighbor-joining tree was constructed using Mega 6.05. 8 most abundance (>1%) *arsM* PSCs with 26 representative *arsM* sequences of each order from the database were selected and a neighbor-joining tree was also constructed using Mega 6.05. Heat maps and clustering analyses of *arsM* PSCs were generated with the R-package (v.3.2.4), which showed the relative abundances of the 23 *arsM* PSCs.

### Statistical Analyses

Statistical analyses were performed with the use of SPSS 20.0 software (SPSS Inc., Chicago, IL). The significant differences in all the measured variables between the composting piles were tested by two-way analysis of variance ANOVA followed by Tukey’s test. A p-value less than 0.05 was considered to be significant. Bivariate correlations were conducted to estimate the link among different parameters.

### Sequence accession

The 16S rRNA gene sequences reported in this paper has been deposited in the National Center for Biotechnology Information Sequence Read Archive (SRA) (BioProject accession number PRJNA315475). Further details on the methods used in this study are included in the Supporting Information.

## Additional Information

**How to cite this article**: Zhai, W. *et al*. Arsenic Methylation and its Relationship to Abundance and Diversity of **arsM** Genes in Composting Manure. *Sci. Rep.* **7**, 42198; doi: 10.1038/srep42198 (2017).

**Publisher's note:** Springer Nature remains neutral with regard to jurisdictional claims in published maps and institutional affiliations.

## Supplementary Material

Supplementary Information
