# Supplementary material for: Arsenic Methylation and its Relationship to Abundance and Diversity of arsM Genes in Composting Manure
Source: Sci Rep. 2017 Mar 7;7:42198. doi: 10.1038/srep42198 (PMC5339872; doi:10.1038/srep42198)
Supplement: Supplementary Information [file srep42198-s1.pdf]

Supplementary Information

Arsenic Methylation and its Relationship to Abundance and Diversity of *arsM* Genes in Composting Manure

Weiwei Zhai[1], Mabel T. Wong[2], Fei Luo[2], Muhammad Z. Hashmi[3], Xingmei Liu[1], Elizabeth A. Edwards[2], Xianjin Tang[1,2,*], Jianming Xu[1]

[1]Institute of Soil and Water Resources and Environmental Science, College of Environmental and Resource Sciences, Zhejiang Provincial Key Laboratory of Agricultural Resources and Environment, Zhejiang University, Hangzhou 310058

[2] Department of Chemical Engineering and Applied Chemistry, University of Toronto, Toronto, M5S 3E5, Canada

[3]Department of Meteorology, COMSATS Institute of Information Technology, Islamabad Campus, Park Road, ChakShahzad, Islamabad, Pakistan

[*]**Corresponding author**: Dr. Xianjin Tang

Tel.: +86-571-8898-2483; Fax: +86-571-8898-2069

E-mail: xianjin@zju.edu.cn

Sampling

The whole composting process lasted for 60 days, and sampling occurred on day 1, 5, 15, 25, 35, 45 and 60. Half of the samples was immediately stored at -80 ℃ for later DNA extraction, while the remainder half was freeze-dried, ground, and filtered through a 0.25 mm Nylon sieve for further arsenic concentration and other chemical analyses. Temperatures at different locations within the piles, including the surface (20 cm to top), center, and bottom (20 cm from bottom) and ambient air were recorded every two days with a mercury-in-glass thermometer. Moisture content was determined on fresh samples by mass loss after drying at 105 ℃ for 24 h. *Eh* and pH were measured using an IQ 150 pH/mV/Temperature system (Aozuo Ecology Instrumentation Ltd., Beijing, China). The C/N ratio was measured with a CNS analyzer (Vario Max CNS, ElementarAnalyseSysteme, Germany) on freeze-dried samples. Available NH_4^+ were determined in 1 MKCl extracts (1:5 w/v) using the colorimetric methods.

Analysis of total As by atomic fluorescence spectrometry (AFS)

For the analysis of total As content, 1.0 g freeze-dried samples was digested with 10 mL 1:1 aqua regia at temperature of 100 ℃ for 2 h with water bath. Solution was cooled and diluted to 50 mL and then total As was determined using an Atomic fluorescence spectrometer (AFS-9130, Beijing Jitian Instrument Company, China).The detection limit for As was 0.05 μg L^{-1}. A soil reference (GBW 07429) purchased from the National Research Centre of China was used to determine the accuracy of the analytical method. The recovery at 95.5±5.8 % (n=3) proved that the method for detection of total As was credible.

Analysis of methylated As by HPLC-ICP-MS

A High Performance Liquid Chromatography Coupled with Inductively Coupled

Plasma Mass Spectrometry (HPLC-ICP-MS,NEXION300XX, PerkinElmer, Inc., USA) was used to measure the concentrations of As species. The freeze-dried samples were first extracted using 10 mL orthophosphoric acid (2%) via ultrasonic extraction. After the ultrasonic extraction, the suspensions were centrifuged, and then filtered through 0.22 μm membrane syringe filters. The filtered samples were kept frozen (-20 ℃) for further analysis. HPLC separation of As species was performed on an anion exchange PRP X-100 HPLC column (250 × 4.1 mm I.D., 10 μm, Hamilton, Reno, NV).The mobile phase consisted of 8 mM $(NH_4)_2HPO_4$ and 2 mM NH_4NO_3 at a pH 6.2 and run isocratically at a flow rate of 1 mL min^{-1} for the entire 10 min. These eluents were prepared from reagent grade salts and 18 MΩ-cm deionized water. The mobile phase was also filtered through 0.22 μm membrane syringe filter anddegassed in an ultrasonic bath. Inductively-Coupled Plasma Mass Spectrometry (ICP-MS) was used to quantify the arsenic species at m/z (mass/charge) 75 corresponding to As using the standard procedures.The ICP-MS was set up in the He gas collision mode and operated with RF power of 1350 W, and a pneumatic nebulizer and cyclonic spray chamber were used. For HPIC-ICP-MS, the injection volume was 20 μL for all standards and samples. The calibration curves were generated by running a three-point calibration with mixed standard solutions for each As species. The concentrations of theAs species were calculated by integrating the corresponding chromatogram peak areas. Each sample was repeated three times with three HPLC injections.

Determination of bacterial 16S rRNA and *arsM* genes copy numbers by qPCR

Copy numbers of bacterial 16S rRNA gene in the compost samples were estimated by quantitative PCR (qPCR) using primers 1369F (5’-CGGTGAATACGTTCYCGG) and 1492R (5’- GGWTACCTTGTTACGACTTT)[1].

PCR reaction mixtures contained 1 μL DNA template (after 10-fold dilution of the DNA extracts of compost samples, 1~50 ng), 0.5 μL of each 10 μM primer pair, 12.5 μL 2×SYBR Green qPCR Super-Mix-UDG with Rox (Invitrogen Con., Shanghai, China), and RNase-free Water (Ambion, USA) for a total volume of 25 μL. PCR amplification was performed with ABI 7500 thermal cycler (Applied Biosystems, Darmstadt, Germany) with the following conditions: initial denaturation at 95 ℃ for 5 min, and 40 cycles of 95 ℃ for 30 s, 55 ℃ for 20 s, 60 ℃ for 30 s, followed by a final extension at 72 ℃ for 10 min. The bacterial standard template DNA was prepared by PCR amplified 16S rRNA using bacteria-specific primer pair (1369F/1492R). The PCR amplicons were ligated into pMD® 18-T vector (TaKaRa Bio Inc., Japan) and the resultant plasmids were transformed into *E.coli* JM109 cells. Plasmid DNA was extracted and the concentration was measured with ND-2000 UV-Vis spectrophotometer (NanoDrop Co., USA). These plasmids were then sequenced and used as the calibration standard for qPCR.

Copies of the *arsM* gene were also determined by qPCR. A 347-bp fragment was amplified using degenerate primers *arsM*F (5’- TCYCTCGGCTGCGGCAAYCCVAC) and *arsM*R (5’- CGWCCGCCWGGCTTWAGYACCCG) designed by *Jia et al*[2] The reactions were performed on ABI 7500 thermal cycler (Applied Biosystems, Darmstadt, Germany) in 25 μL-reaction mixtures containing 1 μL DNA template (after 10-fold dilution of the DNA extracts of compost samples, 1~50 ng), 1 μL of each 10 μM primer pair, and 12.5 μL SYBR Premix Ex TaqTM (Takara Bio Inc., Japan). PCR amplification was performed with the following conditions: initial denaturation at 95 ℃ for 5 min, and 40 cycles of 95 ℃ for 15 s, 60 ℃ for 20 s, 72 ℃ for 30 s, followed by a final extension at 72 ℃ for 7 min. The specificity of the amplification was assessed by melting curve analysis[3]. Standard plasmids prepared by

ligating PCR amplicons into pMD® 18-T vector (TaKaRa Bio Inc., Japan) were transformed into E.coli JM109 cells. And standard curves were prepared via serial dilutions of a known copy number of linearized plasmids.

16SrRNA gene sequencing

The V6-V8 hypervariable region 16S rRNA gene was amplified using universal primers 926F (AAA CTY AAA KGA ATT GAC GG) and 1392R (ACG GGC GGT GTG TRC) with the incorporation of multiplex barcodes[4, 5]. The total volume of the PCR reaction mixture was 50 μL, which consisted of 1 μL DNA template, 0.5 μL of each primer, 5 μLdNTP mixture, 5 μL of 10× Ex Taq buffer (TaKaRa Bio Inc., Japan), 0.25 μL of Ex Taq DNA polymerase (TaKaRa Bio Inc., Japan), and 37.75 μL of Milli-Q water. Two rounds of PCR were performed, with the condition for the first PCR as follows: 94 °C for 3 min; 5 cycles of 94 ℃ for 30 s, 45 ℃ for 20 s and 65 ℃ for 30 s; then 25 cycles of 94 ℃ for 20 s, 55 ℃ for 20 s, and 72 ℃ for 30 s; and a final elongation at 72 ℃ for 5 min. The conditions of the second round PCR were as follow: 95 ℃ for 30 s; 5 cycles of 95 ℃ for 15 s, 55 ℃ for 15 s, 72 ℃ for 30 s; and a final elongation at 72 ℃ for 5 min. The PCR amplicons were purified with a SanPrep Column DNA Gel Extraction Kit (Sangon Biotech, Shang Hai, China), quantified by a Qubit® 2.0 Fluorometer (Invitrogen, Q32866), and then sent for sequencing using IlluminaMiseq sequencing platform (Miseq, Illumina Inc., USA) at Zhejiang Institute of Microbiology, China.

Output sequences (302,994) were quality checked by filtering low quality reads using PRINSEQ (v. 0.19.5) and chimeric sequences using UCHIME package with default settings. After the removal of barcode and primers, the remaining 229,807 sequences were then clustered into 41,339 OTUs at 97% sequence identity by UCLUST (v1.1.579). Taxonomic assignments were performed with the ribosomal

database project (RDP) classifier with a confidence cutoff at 0.8.Sequencing coverage, Shannon-Wiener index, Chao1, and ACE indices were calculated using MOTHUR (v 1.27).

Construction of the *arsM* Clone Library

Four samples (15-MC1, 15-MC2, 60-MC1 and 60-MC2) were selected for the construction of *arsM* gene clone libraries with primers *arsM*F: *arsM*R. *ArsM* amplicons were generated from a 25 μL PCR reaction mixture contained: 2.5 μL 10 × PCR buffer, 2 μLdNTP, 1 μL each 10 μM primer pair, 0.5 μL Ex TaqTM (Takara Bio Inc., Japan), 1μL DNA template (<500 ng), and 18 μL PCR grade water. Optimized PCR thermal cycling parameters were the following: 95 ℃ denatured 10 min, followed by 40 cycles at 95 ℃ for 30 s, 60 ℃ for 45 s, 72 ℃ for 1 min; after these cycles, the reaction mixtures were further incubated at 72 ℃ for 10 min. The PCR products were checked by 2% agarose gel electrophoresis and purified with QIA-quick® Gel Extraction Kit according to the manufacturer's protocol. The purified PCR amplicons were then were ligated into the pMD® 18-T vector (Takara Bio Inc., Japan) and then transformed into *E. coli JM109*. Positive clones (about 100 clones per library) were selected randomly from these clone libraries and sequenced by ABI 3100 Capillary Sequencer (ELIM Biopharm, Hayward, CA, USA). Output sequences (422) were quality checked by filtering low quality reads using MOTHUR program and chimeric sequences using UCHIME package with default settings[6, 7]. After the removal of barcode and primers, the remaining sequences were then clustered into 23 PSCs at 89% sequence identity by UCLUST (v1.1.579).

Supplementary Table S1 Physical and chemical properties of compost samples

Sample	Days	Moisture Content (%)	C/N ratio	pH	Eh (mV)	NH^{4+}-N (mg kg^{-1}dw)	Organic matter (% dw)
MC1	1	64.2	25.3	6.9	192.4	6470.1	63.9
	5	62.0	23.5	4.2	165.5	11204.5	60.9
	15	54.0	22.2	3.4	213.5	7674.5	57.5
	25	47.1	18.5	5.1	141.2	4804.0	50.8
	35	45.1	16.7	5.6	61.9	3481.2	53.0
	45	44.6	15.4	6.8	26.6	1863.0	52.1
	60	41.3	13.1	5.9	64.6	2023.4	42.2
MC2	1	63.4	25.4	6.9	155.8	9427.5	57.2
	5	61.2	24.3	4.2	160.6	11498.2	57.8
	15	53.4	23.7	3.5	204.6	8100.4	56.0
	25	49.9	19.1	5.3	124.7	4149.0	55.1
	35	46.3	15.8	6.6	38.4	3100.4	50.2
	45	46.8	14.4	6.1	45.9	3304.7	49.8
	60	44.7	12.3	6.0	59.9	1754.3	40.5

Supplementary Table S2 ANOVA test analysis of measured As concentrations and *arsM* gene copies in MC1 and MC2

Day	Total As (mg kg^{-1}dw)		MMA (mg kg^{-1}dw)		DMA (mg kg^{-1}dw)		Total methylated As (MMA+DMA, mg kg^{-1}dw)		*ArsM* gene copies (*$10^9 g^{-1}$dw)	
	MC1	MC2	MC1	MC2	MC1	MC2	MC1	MC2	MC1	MC2
1	1270.1±61.5bc	1240.3±66.9c	6.1±0.6e	4.9±1.9e	29.6±3.7g	36.8±2.7g	35.7±4.3f	41.7±0.4f	0.10±0.01h	0.07±0.01h
5	1498.8±184.6abc	1535.9±65.6abc	30.1±6.5de	27.5±0.9de	51.4±1.4g	54.3±0.7g	81.5±7.9f	81.8±1.7f	0.52±0.01h	0.37±0.02h
15	1610.3±261.8abc	1692.6±33.5abc	167.7±3.1b	211.7±14.0a	126.3±6.4f	259.6±43.2e	294.0±9.5e	471.3±29.2d	1.72±0.03g	1.99±0.02g
25	1927.9±158.0a	1626.1±236.3abc	174.6±20.7b	171.6±3.4b	224.9±7.4 e	412.0±3.6d	399.5±13.4d	583.6±0.2bc	2.90±0.29f	3.82±0.12de
35	1831.0±352.3a	1728.9±104.5abc	160.6±7.8b	103.4±8.9c	465.2±16.0cd	455.4±26.0cd	625.8±23.7abc	558.8±34.9c	3.66±0.38ef	4.61±0.44 cd
45	1862.8±163.4a	1638.9±248.0abc	106.6±2.8c	101.6±1.2c	544.0±11.1b	505.5±26.4bc	650.7±8.3ab	607.1±27.6abc	5.01±0.47bc	5.57±0.24b
60	1800.1±68.4ab	1720.9±180.2abc	42.5±2.7d	27.7±3.8de	619.9±10.2a	569.5±19.5ab	662.4±12.9a	597.2±23.2abc	6.54±0.52a	7.11±0.52a

Different letters following concentrations indicate significant ($p \leqslant 0.05$) differences between MC1 and MC2 and between different composting days for each analysis.

SupplementaryTable S3 Results of 16s rRNA amplicon sequencing including number of reads, number of OTU, and alpha diversity indices

Sample	Days	No. of reads	No. of OTUs	Shannon	ACE	Chao1	Coverage
MC1	1	28074	4591	5.90	26067	14281	0.89
	15	22861	6966	6.91	49030	24996	0.77
	25	22873	6794	6.91	47970	24215	0.78
	45	18930	6045	7.17	45213	22399	0.76
	60	19201	6653	7.43	52127	25265	0.74
MC2	1	27073	4621	5.92	25559	14786	0.88
	15	23810	7006	6.89	49206	24587	0.78
	25	24705	7234	7.14	51183	26360	0.78
	45	19414	6489	7.43	54720	25509	0.75
	60	22866	7529	7.47	56014	27879	0.75

Supplementary Table S4 27 representative16s OTUs with selected best match references sequences used in the Neighbor-joining tree. A custom database of microorganisms contained *arsM* genes was constructed by downloading all 16S rRNA gene sequences from NCBI. All 16S rRNA gene sequences of samples were checked against the database and sequence that has a similarity≥95% and reads ≥ 10 at one time point were retained.

OTU	Reference sequences of known functional bacteria	Ident.(%)	Numbers of reads										Taxonomic
			MC1					MC2					
			1d	15d	25d	45d	60d	1d	15d	25d	45d	60d	
**4319**	***Methanosarcinathermophila TM-1***	99	0	0	0	2	2	0	0	0	5	1	Methanosarcinales (Euryarchaeota)
**34877**	***Sphaerobacter thermophiles DSM 20745***	99	0	0	9	43	46	0	4	36	46	117	Sphaerobacterales (Chloroflexi)
**35506**		97	0	0	2	19	13	0	1	7	26	57	
**35326**		96	0	1	8	13	8	0	5	14	12	27	
**205**	***Streptomyces sp. GSRB54***	97	0	0	0	0	29	0	0	0	21	4	Streptomycetales (Actinobacteria)
**15490**	***Mycobacterium parascrofulaceum strain ATCC BAA-614***	97	1	0	0	2	70	0	0	0	109	2	Corynebacteriales (Actinobacteria)
**10791**		96	0	0	0	0	43	0	0	0	68	7	
**16423**		96	0	0	0	0	48	0	0	0	1	7	
**9513**	***AlkaliphilusoremlandiiOhILAs***	96	0	2	2	13	3	0	2	8	5	12	Clostridiales (Firmicutes)
**4401**	***Paenibacillus sp. A3***	96	0	0	0	13	6	0	0	4	1	15	Bacillales (Firmicutes)
**1947**	***Bacillus sp. FJAT-21945***	97	0	11	19	36	45	0	17	73	166	126	
**20257**		95	0	2	5	183	9	0	1	81	7	3	
**4359**		95	0	1	2	4	153	0	0	2	85	2	
**15855**		95	1	54	15	38	35	2	28	8	21	6	

OTU	Reference sequences of known functional bacteria	Ident.(%)	Numbers of reads										Taxonomic
			MC1					MC2					
			1d	15d	25d	45d	60d	1d	15d	25d	45d	60d	
**11005**	*Viridibacillusarvi*	96	11	0	0	0	0	19	0	0	0	0	Bacillales (Firmicutes)
**4960**		95	210	1	4	4	0	267	2	4	1	1	
**8631**		95	0	0	3	3	21	0	4	28	4	4	
**8520**	*Bacillus sp. 1NLA3E*	95	0	29	18	0	2	0	9	21	10	9	
**1953**	*Thioalkalivibriosulfidophilus HL-EbGr7*	96	0	10	32	34	36	0	19	29	38	65	Chromatiales (γ-proteobacteria)
**4352**		95	0	57	20	13	31	0	95	18	7	11	
**8822**		95	0	8	65	2	5	0	12	6	32	31	
**7182**	*Amycolatopsismediterranei U32*	95	2	0	11	329	74	0	2	45	82	529	Pseudonocardiales (Actinobacteria)
**28850**	*Thiobacillusdenitrificans ATCC 25259*	95	1	7	33	36	21	0	13	45	22	28	Hydrogenophilales (β-proteobacteria)
**31542**		95	2	0	6	2	2	1	0	29	2	2	
**30525**		95	0	0	1	3	4	0	2	1	7	9	
**15267**	*Sulfuricelladenitrificans skB26*	95	0	0	0	10	8	0	0	0	4	2	Sulfuricellales (β-proteobacteria)
**14561**		95	0	1	1	2	5	0	1	3	5	4	

Supplementary Table S5 The database of species containing a known *arsM* sequence (Extracted from NCBI)[a]

NO.	Species	*arsM* gene (protein) access number	16S rRNA gene access number	NO.	Species	*arsM* gene access number	16SrRNA gene access number
1	***[Clostridium] purinilyticum putative***	LN679998.1	/	22	***Clostridium sp. BXM***	AIM18906.1	JN092128.1
2	***[Clostridium] sordellii VPI 9048***	NZ AQGJ01000162.1	/	23	***Conexibacterwoesei DSM 14684***	NC 013739.1	/
3	***AlkaliphilusoremlandiiOhILAs***	NC 009922.1	NR 074435.1	24	***Criblamydiasequanensis***	CDR35322.1	DQ124300.1
4	***Amycolatopsismediterranei S699***	NC 018266.1	/	25	***Cupriavidusmetallidurans CH34***	NC 007973.1	NR 074704.1
5	***Amycolatopsismediterranei U32***	CP002000.1	NR 102906.1	26	***Cytophagahutchinsonii ATCC 33406***	NC 008255.1	NR 102866.1
6	***Bacillus decisifrondis***	KOS62122.1	/	27	***Dehalococcoidesethenogenes 195***	NC 002936.3	DQ834686.1
7	***Bacillus sp. 1NLA3E***	NC 021171.1	FJ851432.1	28	***Dehalococcoidesmccartyi 195***	NC 002936.3	NR 074116.1
8	***Bacillus sp. FJAT-18043***	KQL19469.1	KP268082.1	29	***Dehalococcoidesmccartyi BTF08***	NC 020387.1	NR 102515.1
9	***Bacillus sp. FJAT-21945***	KOP82460.1	KM978195.1	30	***Dehalococcoidesmccartyi DCMB5***	NC 020386.1	/
10	***Bacteroidesfragilis 3-1-12***	EFR52264.1	/	31	***Dehalococcoidesmccartyi GY50***	NC 022964.1	/
11	***Bellilineacaldifistulae***	KPL76409.1	AB243672.1	32	***Dehalococcoidesmccartyi IBARAKI***	BAS32294.1	/
12	***CandidatusEntotheonella sp. TSY1***	ETX01300.1	/	33	***Dehalococcoides sp. BAV1***	NC 009455.1	AY165308.1
13	***CandidatusEntotheonella sp. TSY2***	ETX04452.1	/	34	***Dehalococcoides sp. CBDB1***	NC 007356.1	NR 074115.1
14	***CandidatusMethanoperedens sp. BLZ1***	KPQ44095.1	/	35	***Desulfatitalea sp. BRH-c12***	KJS30638.1	/
15	***CandidatusMethanoplasmatermitum putative***	AIZ56307.1	/	36	***Deltaproteobacteria bacterium SG8-13***	KPJ76183.1	/
16	***CandidatusMethanoplasmatermitum putative(2)***	AIZ57293.1	/	37	***Desulfitobacteriumhafniense DCB-2***	NC 011830.1	NR 074996.1
17	***CandidatusSolibacterusitatus Ellin6076***	NC 008536.1	NR 074351.1	38	***Desulfitobacteriumhafniense Y51***	NC 007907.1	/
18	***CandidatusThiomargaritanelsonii***	KHD05161.1	/	39	***Desulfococcusoleovorans Hxd3***	NC 009943.1	NR 075012.1
19	***Chitinophagapinensis DSM 2588***	NC 013132.1	NR 074566.1	40	***Desulfohalobiumretbaense DSM 5692***	NC 013223.1	NR 074907.1
20	***Chlorobiumphaeobacteroides BS1***	NC 010831.1	NR 074363.1	41	***Draconibacterium sp. JN14CK-3***	KJF44230.1	/
21	***Clostridium pasteurianum DSM 525***	AJA50588.1	/	42	***Gammaproteobacteria bacterium NRL1***	ALP54027.1	/

43	***Gemmatimonasaurantiaca T-27***	NC 012489.1	NR 074708.1	67	***Methanosarcinabarkeri 227***	NZ CP009530.1	/
44	***Geobacilluskaustophilus HTA426***	NC 006510.1	NR 074989.1	68	***Methanosarcinabarkeri 3***	NZ CP009517.1	/
45	***Geobacillus sp. Y4.1MC1***	NC 014650.1	/	69	***Methanosarcinabarkeri CM1***	NZ CP008746.1	/
46	***Geobacillus sp. A8***	EQB94645.1	KM192159.1	70	***Methanosarcinabarkeri MS***	NZ CP009528.1	/
47	***Geobacillus sp. CAMR5420***	KDE50131.1	/	71	***Methanosarcinabarkeri str. Fusaro***	NC 007355.1	NR074253.1
48	***Geobacillusstearothermophilus NUB3621***	EZP76693.1	/	72	***Methanosarcinabarkeri str. Wiesmoor***	NZ CP009526.1	/
49	***Geobacillusthermoglucosidasius***	ALF10841.1	/	73	***Methanosarcinahoronobensis HB-1***	NZ CP009516.1	/
50	***Geobactermetallireducens GS-15***	NC 007517.1	NR 075011.1	74	***Methanosarcinalacustris Z-7289***	NZ CP009515.1	
51	***Gloeobacterkilaueensis JS1***	NC 022600.1	NR 121745.1	75	***Methanosarcinamazei C16***	NZ CP009514.1	/
52	***Halalkalicoccusjeotgali B3***	NC 014297.1	NR 044072.1	76	***Methanosarcinamazei Go1***	NC 003901.1	NR074221.1
53	***Halobacteriumsalinarum R1***	NC 010368.1	NR 074204.1	77	***Methanosarcinamazei LYC***	NZ CP009513.1	/
54	***Halobacterium sp. DL1***	NZ CP007060.1	/	78	***Methanosarcinamazei S-6***	NZ CP009512.1	/
55	***Halobacterium sp. NRC-1***	NC 001869.1	AB663363.1	79	***MethanosarcinamazeiSarPi***	NZ CP009511.1	/
56	***Halorubrumlacusprofundi ATCC 49239***	NC 012029.1	/	80	***Methanosarcinamazei WWM610***	NZ CP009509.1	/
57	***Imtechellahalotolerans K1***	EID71665.1	/	81	***Methanosarcina sp. Kolksee***	NZ CP009524.1	/
58	***Methanobacterium lacus***	NC 015216.1	/	82	***Methanosarcina sp. MTP4***	NZ CP009505.1	/
59	***Methanobacterium lacus strain AL-21***	NC 015216.1	NR 102889.1	83	***Methanosarcina sp. WH1***	NZ CP009504.1	/
60	***Methanobacteriumpaludis strain SWAN1***	NC 015574.1	NR 133895.1	84	***Methanosarcina sp. WWM596***	NZ CP009503.1	
61	***Methanoculleusmarisnigri JR1***	NC 009051.1	KM041240.1	85	***Methanosarcinathermophila TM-1***	NZ CP009501.1	NR 118372.1
62	***Methanohalobiumevestigatum Z-7303***	NC 014253.1	NR 074251.1	86	***Methanosarcinavacuolata Z-761***	NZ CP009520.1	/
63	***Methanolaciniapetrolearia DSM 11571***	NC 014507.1	U76631.1	87	***Microcystisaeruginosa PCC 7806***	AEK49145.1	KF286992.1
64	***Methanosaetaconcilii GP6***	NC 015416.1	NR104707.1	88	***Mycobacterium parascrofulaceum ATCC BAA-614***	EFG78363.1	NR 118110.1
65	***Methanosaetaharundinacea 6Ac***	NC 017527.1	KM408632.1	89	***Microcystisaeruginosa TAIHU98***	ELP56516.1	/
66	***Methanosarcinaacetivorans C2A***	NC 003552.1	/	90	***Paenibacillus borealis***	AIQ61043.1	AB073364.1

91	***Paenibacillusdurus***	AIQ11909.1	AB073195.1	112	***Pontibacter sp. BAB1700***	EJF08553.1.	/
92	***Natronomonasmoolapensis 8.8.11***	NC 020388.1	AY498645.2	113	***Rhodopseudomonaspalustris CGA009***	NC 005296.1	/
93	***Paenibacillusdurus(2)***	AIQ12917.1	/	114	***Rhodopseudomonaspalustris BisB5***	NC 007958.1	/
94	***Paenibacillusgraminis***	AIQ71140.1	AB428571.1	115	***Rhodopseudomonaspalustris HaA2***	NC 007778.1	/
95	***Paenibacilluspolymyxa***	KOS00701.1	/	116	***Rhodopseudomonaspalustris TIE-1***	NC 011004.1	NR 103926.1
96	***Paenibacilluspolymyxa M1***	NC 017542.1	KF583677.1	117	***Rubrivivaxbenzoatilyticus JA2***	EGJ09640.1.	/
97	***Paenibacillussabinae T27***	AHV97393.1	/	118	***Salinibacterruber DSM 13855***	NC007677.1	/
98	***Paenibacillus sp. A3***	KPV55612.1	HG003584.2	119	***Sporolactobacilluslaevolacticus DSM 442***	EST12011.1.	/
99	***Paenibacillus sp. D9***	KKC46061.1	GQ368737.2	120	***Streptomyces roseosporus NRRL 11379***	NZ ABYX02000001.1	/
100	***Paenibacillus sp. FSL H7-0357***	AIQ18494.1.	/	121	***Streptomyces sp. GSRB54***	BAO65696.1.	AB856548.1
101	***Paenibacillus sp. HGF7***	EGL18649.1	/	122	***Sulfuricelladenitrificans skB26***	NC 022357.1	NR 121695.1
102	***Paenibacillus sp. IHB B 3084***	ALP37443.1.	/	123	***Paenibacillus sp. Soil766***	KRE96462.1.	/
103	***Paenibacillus sp. IHB B 3415***	KHL96773.1.	/	124	***Synechocystis sp. PCC 6803***	ADL59372.1.	AY224195.1
104	***Symbiobacteriumthermophilum IAM 14863***	NC 006177.1	/	125	***Syntrophusaciditrophicus SB***	NC 007759.1	/
105	***Paenibacillus sp. VKM B-2647***	KIL39348.1.	/	126	***Thermacetogeniumphaeum DSM 12270***	NC 018870.1	NR 074723.1
106	***Pedobacter sp. BAL39***	EDM36947.1.	/	127	***Thermosediminibacteroceani DSM 16646***	NC 014377.1	NC 014377.1
107	***Pelobactercarbinolicus DSM 2380***	NC 007198.1	NR 075013.1	128	***Thioalkalivibriosulfidophilus HL-EbGr7***	NC 011901.1	NR 074692.1
108	***Pelobacterpropionicus DSM 2379***	NC 008609.1	NR 074975.1	129	***Thiobacillusdenitrificans ATCC 25259***	NC 007404.1	NR 074417.1
109	***Pelotomaculumthermopropionicum SI***	NC 009454.1	NR 074685.1	130	***Viridibacillusarvi***	KOO51828.1.	AY266991.1
110	***Sphaerobacterthermophilus strain DSM 20745***	NC 013523.1	AJ420142.1				
111	***Thermanaerovibrioacidaminovorans DSM 6589***	NC 013522.1	NR 074520.1				

[a]The accession numbers of *arsM* gene or protein and 16S rRNA gene were listed in the table, while the 16S rRNA genes of some species were not found.

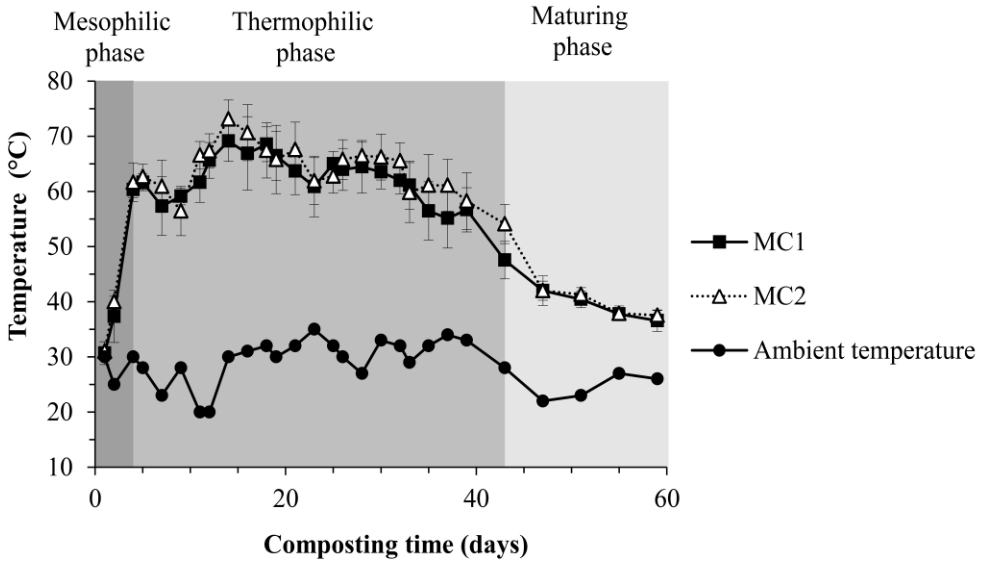

Supplementary Figure S1 Temperature profiles of two compost piles (MC1; MC2). The composting pile temperatures went through mesophilic (day 0-4), thermophilic (day 5-42), and maturing phases (day 43-60). MC1 contained 1,200 kg pig manure and 600 kg sawdust. MC2 contained 1,200 kg pig manure and 600 kg sawdust mixed with burned rice straw.

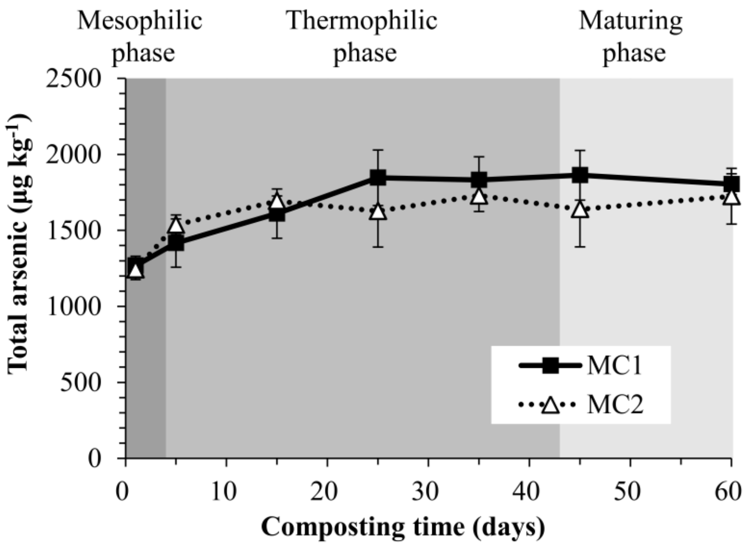

Supplementary Figure S2 Change of total arsenic concentration versus time during pig manure composting. Error bars represent the standard error of 3 replicate analysis of a composite sample. A composite sample was made up of 10 subsamples from different locations in compost piles.

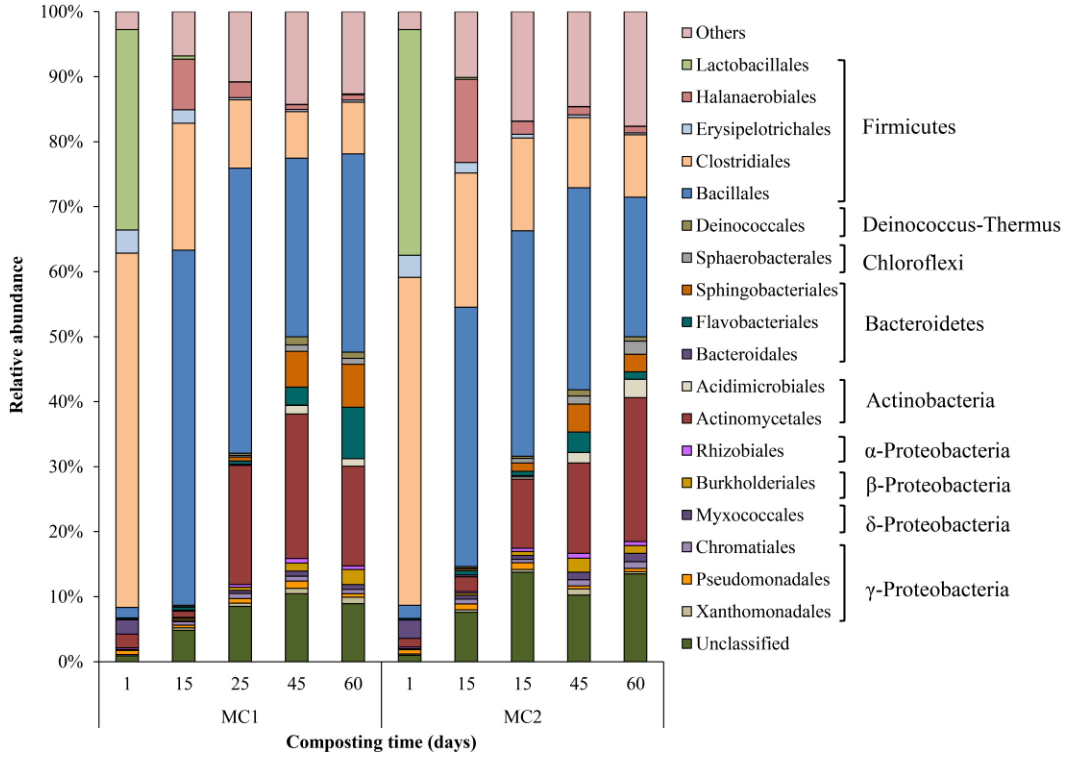

Supplementary Figure S3 Microbial community composition of each sample at the order level. Phylum or Class level is indicated in bracket. The predominant 9 phylum/class and 18 orders are shown.

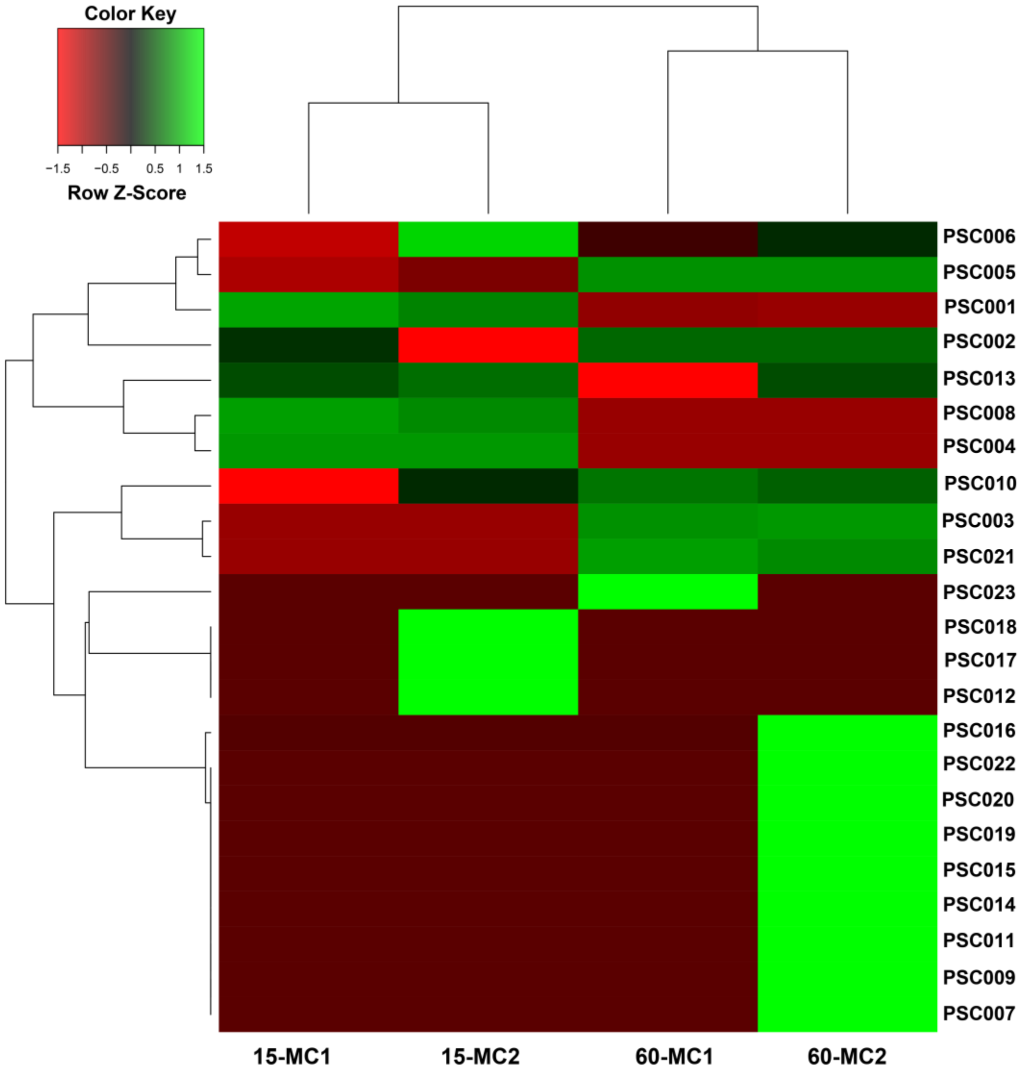

Supplementary Figure S4 Abundance of microbial *arsM* genes in four samples (15-MC1 15-MC2, 60-MC1 and 60-MC2) based on partial sequences clones (PSCs). Complete linkage clustering of four samples was calculated by the composition and relative abundance of *arsM* genes. 23 PSCs are shown in the heat map. Scales of green, black, and red indicated the relative abundance of each PSC in different samples.

References

1. Sheng, R. *et al.* Bacterial succession in paddy soils derived from different parent materials. *J. Soils. Sed*. **15(4)**, 982-992 (2015).

2. Jia, Y. *et al.* Microbial arsenic methylation in soil and rice rhizosphere. *Environ. Sci. Technol*. **47(7)**, 3141-3148 (2013).

3. Dias, A. C. *et al.* Abundance and genetic diversity of *nifH* gene sequences inanthropogenically affected Brazilian mangrove sediments. *Appl. Environ. Microbiol*. **78(22)**, 7960-7967 (2012).

4. Kozubal, M. A. *et al.* Microbial iron cycling in acidic geothermal springs of yellowstone national park: integrating molecular surveys, geochemical processes, and isolation of novel fe-active microorganisms. *Front. Microbiol.* 154 (2012).

5. Berdugo-Clavijo, C. & Gieg, L. M. Conversion of crude oil to methane by a microbial consortium enriched from oil reservoir production waters. *Front. Microbiol.* **5**, 197 (2014).

6. Schloss, P. D. & Westcott, S. L. Assessing and improving methods used in operational taxonomic unit-based approaches for 16S rRNA gene sequence analysis. *Appl. Environ. Microbiol*. **77(10)**, 3219-3226 (2011).

7. Liu, Y. R., Yu, R. Q., Zheng, Y. M. & He, J. Z. Analysis of the microbial community structure by monitoring an Hg methylation gene (hgcA) in paddy soils along an Hg gradient. *Appl. Environ. Microbiol*. **80(9)**, 2874-2879 (2014).
